# A deep learning approach for detecting liver cirrhosis from volatolomic analysis of exhaled breath

**DOI:** 10.3389/fmed.2022.992703

**Published:** 2022-09-29

**Authors:** Mikolaj Wieczorek, Alexander Weston, Matthew Ledenko, Jonathan Nelson Thomas, Rickey Carter, Tushar Patel

**Affiliations:** ^1^Digital Innovation Lab, Mayo Clinic, Jacksonville, FL, United States; ^2^Department of Transplant, Mayo Clinic, Jacksonville, FL, United States

**Keywords:** deep learning, cirrhosis, volatile organic compound, prediction, breath

## Abstract

Liver disease such as cirrhosis is known to cause changes in the composition of volatile organic compounds (VOC) present in patient breath samples. Previous studies have demonstrated the diagnosis of liver cirrhosis from these breath samples, but studies are limited to a handful of discrete, well-characterized compounds. We utilized VOC profiles from breath samples from 46 individuals, 35 with cirrhosis and 11 healthy controls. A deep-neural network was optimized to discriminate between healthy controls and individuals with cirrhosis. A 1D convolutional neural network (CNN) was accurate in predicting which patients had cirrhosis with an AUC of 0.90 (95% CI: 0.75, 0.99). Shapley Additive Explanations characterized the presence of discrete, observable peaks which were implicated in prediction, and the top peaks (based on the average SHAP profiles on the test dataset) were noted. CNNs demonstrate the ability to predict the presence of cirrhosis based on a full volatolomics profile of patient breath samples. SHAP values indicate the presence of discrete, detectable peaks in the VOC signal.

## Introduction

Cirrhosis of the liver is an advanced stage of disease in which the liver is damaged from scarring or fibrosis as a result of chronic hepatic injury that can arise from conditions such as chronic infection with Hepatitis B or C virus, excess alcohol or other causes (1). Cirrhosis can be classified as compensated or decompensated. A diagnosis of compensated cirrhosis can be challenging as these patients can be asymptomatic and may have normal laboratory or imaging findings.

The presence of cirrhosis can be inferred from clinical, laboratory, radiologic, or elastographic findings, but a liver biopsy represents the gold-standard for diagnosis (2). Cirrhosis is a preneoplastic condition and a major risk factor for hepatocellular carcinoma (HCC) which is the sixth most prevalent cancer and third leading cause of cancer-related death (3). Once diagnosed, close monitoring for progression as well as active surveillance for onset of HCC are essential (4). The onset of complications such as ascites, varices, and hepatic encephalopathy define decompensated cirrhosis, and are associated with a higher risk of death (5).

Liver disease has long been recognized to be associated with detectable changes in a patient’s breath, e.g., fetor hepaticus (6, 7). These result from the presence of diverse range of Volatile Organic Compounds (VOCs), which may consist of byproducts of liver metabolism that are released into the bloodstream and eventually eliminated in the patient’s breath. Importantly, VOCs have been associated with liver cirrhosis (8) and fibrosis (9). Thus, a reliable detection of disease-associated VOC or other metabolites altered by liver damage have the potential for use as a non-invasive test for the diagnosis and monitoring of cirrhosis.

Global volatolomic analyses can be performed on exhaled breath samples by separating and detecting individual VOCs. The approaches for detection of individual VOC are laborious and time consuming, and often require the use of sophisticated equipment. A limitation of several prior breath-based biomarker studies is that they rely on identification of a single VOC such as limonene (10), which may miss more complex signatures of disease. The large number and variability of VOC in exhaled breath have hampered the development of individual breath-based biomarkers for disease. Recognizing the inherent variability and diversity of individual VOCs with biomarker potential, we sought to evaluate approaches for an unbiased global volatolomic profiles as disease biomarkers. For our study, global volatolomic profiling was performed using thermal desorption (TD) with gas chromatography (GC) based separation coupled with field asymmetric ion mobility spectrometry (FAIMS) for biomarker discovery.

Analysis of volatolomic profiles has been greatly aided with the use of artificial intelligence (AI) algorithms such as deep CNNs which can analyze relationships between all detectable compounds represented in a breath sample.

This study builds upon existing techniques to diagnose liver cirrhosis from non-invasive breath samples using an artificial neural network based on TD-GC-FAIMS signal. We demonstrate that cirrhosis results in detectable, quantifiable changes in the volatolomic profile of a patient’s breath. Furthermore, by utilizing Shapley Additive Explanations, we demonstrate a set of volatolomic features that correspond to disease prediction and reflect biomarkers that can be used for the detection of disease without the need to rely on identification of individual VOCs.

## Materials and methods

### Study participants

This prospective study was conducted under a Mayo Clinic institutional review board (IRB) approved protocol and conformed to the ethical guidelines of the Declaration of Helsinki. Informed consent was obtained from study participants in writing. The trial is registered at clinicaltrials.gov (NCT04341012).

All participants in this single-center prospective study were enrolled between September 2019 and March 2020. The study inclusion criteria were the ability to provide informed consent and age greater than 18 years. Healthy volunteers were employees of the hospital who were recruited to participate through word-of-mouth. Exclusion criteria for healthy controls included a history of liver disease. Patients were categorized into groups based on absence or presence of cirrhosis and/or portal hypertension, and of individual complications as determined on clinical bases which included histologic, clinical, laboratory, or imaging features. Participants with non-cirrhotic portal hypertension were excluded.

### Variable definitions

A clinical diagnosis of cirrhosis served as our ground truth training label and reference standard. Cirrhosis was classified as stage I, stage II, or stage III. Stage I was defined as compensated cirrhosis with the absence of varices or other clinical complications. Stage II (compensated) cirrhosis was defined as presence of varices but no other complications. The presence of varices in patients with compensated cirrhosis is a prognostic factor and indicates a higher risk of decompensation. Stage III (decompensated) cirrhosis was defined as the presence of ascites, variceal hemorrhage, or hepatic encephalopathy. Diagnoses of cirrhosis and presence of clinical complication were determined independently by two hepatologists.

### Sample collection

**FIGURE 1:**
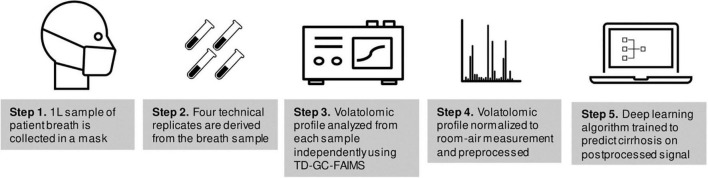
Flow-chart of sample collection and analysis.

A flow-chart of sample collection and volatolomic analysis is shown in Figure 1. Each study participant provided a single breath sample collected using the ReCIVA breath sampler (Owlstone Medical, Cambridge, UK) and passed through thermal desorption tubes to capture VOCs, then separated using high temperatures and GC and passed onto FAIMS (Owlstone Medical, Cambridge, UK), a spectrometry device which separates ions based on size and charge to create a data matrix that represents a volatolomic profile of the breath sample (11). FAIMS has been used for VOC detection in many settings (12–18).

Data collection using this approach (TD-GC-FAIMS) has been described previously (19). Each study participant provided a breath sample totaling to 1-L of exhaled air onto Bio-Monitoring TD tubes (Markes International, South Wales, United Kingdom). Samples were divided into four technical replicates which were derived from the same 1 L breath sample and were collected simultaneously on four separate collection tubes by the collection mask. Samples were collected by a trained technician (J.T.) after patients had fasted at least 4 h from food or drink besides water. A subset of 11 samples from 11 individuals (1 sample per individual) had been stored for a period of time exceeding 6 weeks; these samples were excluded from the analysis because the effects of long-term cold storage on breath VOCs are poorly understood (20, 21).

### Analysis of volatile organic compounds

The TD-GC-FAIMS data output was preprocessed to separate the ion intensities from each dispersion field (DF) setting and subtract out environmental VOCs and background current fluctuations using air filter field control blanks. All technical replicates were analyzed independently. The negative and positive ion intensity mesh matrices at each respective DF were combined and outer matrix cells with intensity values below the overall maximum baseline intensity (0.0104 pA) were removed. Compensation field (CF) scan points were limited to between −3 V and + 3 V. In addition, 30 terminal time resolved values, approximately 40 s at the end of the GC run, were removed for each DF data matrix. Data preprocessing was conducted in Matlab version 2019b (MathWorks, Matick, MA).

For purposes of training deep-learning models, outputs were additionally processed by dividing by the maximum value. The signal was downsampled from a 2D to a 1D signal by taking the maximum value for each row. The final output of the workflow was a signal with 3,400 rows. Note that although the signal is sampled into 3,400 discrete values, there are not 3,400 features present in the signal; a single TD-GC-FAIMS peak spans several rows, and the dataset is sparse with many rows having a value of zero. This is analogous to DL prediction based off electrocardiography signal, where a sample of 10,000 points will capture 10–12 discrete peak features (22). During model training, data were randomly augmented with 5% Gaussian random noise.

### Training of the deep learning model

For model evaluation purposes, all samples from 22 individuals (totaling 75 samples), including 17 positive patients (59 samples) and 5 healthy controls (16 samples) were randomly selected and set aside as a test dataset; these samples were excluded from the model development process. The dataset was split at the patient level such that no patients had samples in both the training and test dataset. The 24 individuals (82 samples), which included 18 positive patients (64 samples) and 6 healthy controls (18 samples) were used for training and validating the neural network model. The ground truth label was taken to be the presence of cirrhosis as determined by clinical experts. All results are reported on the test dataset.

**FIGURE 2:**
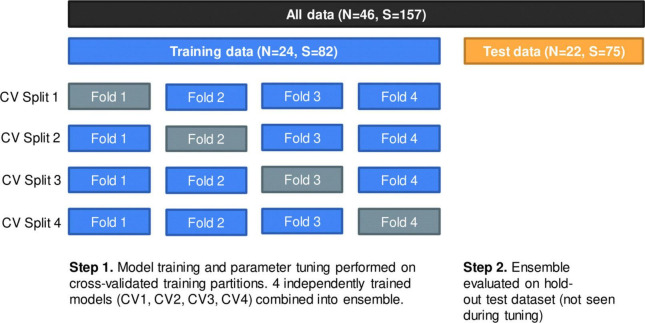
Data partition using group fourfold cross-validation (CV) method. Within training data, each split represents one independently trained model. Models were evaluated on a hold-out test dataset of 22 patients (75 samples).

The 24 individuals were randomly divided into four splits using stratified group fourfold cross-validation; each split consisted of three analysis folds and one assessment fold where the analysis folds were used for training and the assessment fold was used for validating the model. The same cross-validation (CV) split was used for all iterations of hyperparameter tuning. Partitioning was done at the patient level such that no individual had samples in more than a single CV fold. Although partitioning patients into each fold was random, we attempted to preserve the distribution of our outcome with stratification, where at least one healthy patient was represented in each CV split; this was necessary to ensure proper training of the model. Figure 2 displays the data partition; Supplementary Table 2 provides additional details.

### Model development

Several potential deep learning model architectures were evaluated to predict presence of cirrhosis. We selected a custom convolutional neural network (CNN) model for architecture and hyperparameter-tuning process, which outperformed several other models in an initial phase of experimentation (including pretrained ResNet and a custom fully connected deep-neural network).

**FIGURE 3:**
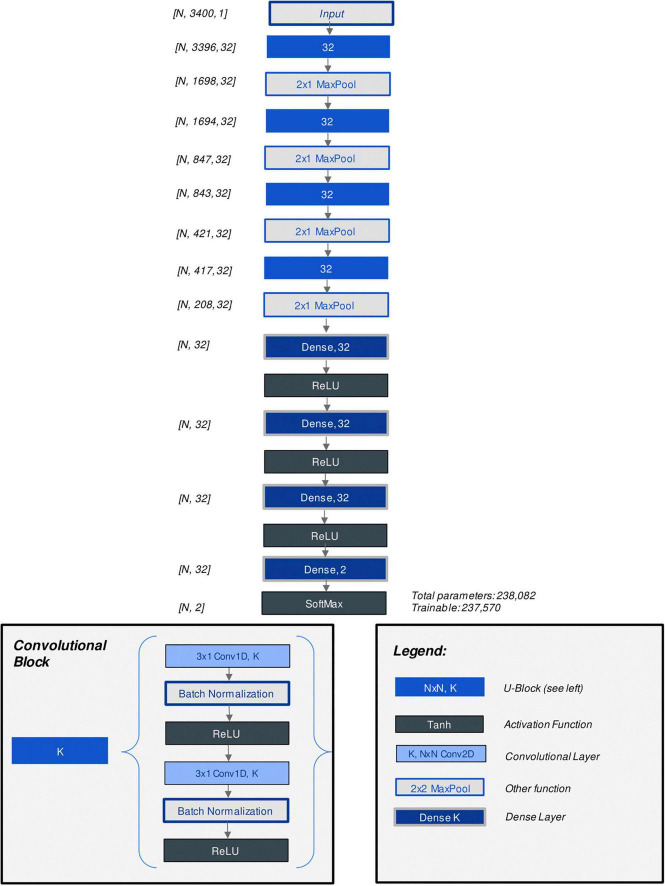
Diagram of custom CNN model architecture.

The CNN model architecture and hyperparameters were optimized through grid search. Parameters which were considered included the number of convolutional layers, number of kernels per layer, number of fully connected (“dense”) layers at the output end of the model, learning rate, batch size as well as alternate methods of augmenting the data to account for data imbalance. Figure 3 displays the architecture of the best-performing CNN model discovered through hyperparameter tuning. Supplementary Table 3 lists all parameters considered (23). Additionally, model architecture and hyperparameter optimization grid search results were summarized with analysis (training) and assessment (validation) accuracy and loss curves in an interactive R markdown document (R version 4.0.3 with Shiny 1.6.0).

The optimal model architecture and hyperparameter configuration was selected by assessing the average highest performing validation accuracy and lowest validation loss across all four CV splits. The best-performing four individual CNN models, one for each corresponding CV split, were combined into an ensemble model by taking the average of model outputs.

Model development with hyperparameter tuning was performed on the Google Cloud Platform (GCP) and was accelerated using 1 Nvidia T4 GPU (16GB RAM).

### Model evaluation

The primary endpoint of this study was diagnosis of liver cirrhosis at the patient level, which was achieved by combining the four individual constituent CNN models from four cross-validation splits (CV1, CV2, CV3, CV4) (i.e., at the dataset level) into an “ensemble” prediction by taking the mean probability across all four models, and then by taking the median value of this ensemble prediction across the 3–4 technical replicates per patient. Additionally, to evaluate the reproducibility of model prediction across multiple technical replicates, the AUC curve and summary metrics are reported at the sample level.

To investigate the algorithm’s ability to discriminate cirrhosis patients from healthy controls, the ensemble model’s predicted probabilities were tabulated by cirrhosis stage and visualized with boxplots.

### Model explainability

Interpretation of AI algorithms is an increasingly important approach to validate their performance and lend insight to the modeling process. To aid in the interpretation of the results of the CNN, we utilized SHapley Additive ExPlanations (SHAP) (RRID:SCR_021362) to determine which features contribute to the detection of liver disease (24). SHAP identifies features which are important in determining the model output by allocating contributions of the model output across input parameters. SHAP was implemented in the SHAP package version 0.39.0 for Python 3.7.8. SHAP values were computed individually for the four CNN models.

SHAP feature importance plots were summarized on the training and test datasets for each CV split with “beeswarm” scatter plots (24). To identify individual compounds from TD-GC-FAIMS which are most important for detecting the presence of liver disease, the five features with the largest magnitude (largest absolute SHAP value) were selected per each instance in the test dataset (75 samples) and were overlayed on the sample’s VOC signal, creating “heatmaps” which identify peaks important for predictions. The heatmaps were visualized with darker red representing the higher number of times the same peak was detected across four constituent models. For each patient, the final ensemble predicted probability was annotated.

### Statistical analysis

Clinical demographics and laboratory test results data were summarized with the median and range for the continuous variables and with the number and percentage of patients for the categorical variables. The Wilcoxon rank sum test for continuous variables and Fisher’s exact test for categorical variables were used to compare demographics between healthy controls and cirrhosis patients; Kruskal-Wallis rank sum test was used to compare laboratory test results between stage I, stage II, and stage III cirrhosis.

Model performance was assessed for the four individual cross-validated models as well as the ensemble model at the sample and patient levels using the area under the receiver operating characteristic curve (AUC), accuracy, sensitivity, specificity, positive predictive value, negative predictive value, and F1 score. A threshold cutoff value of > 0.50 was used to classify a sample or patient as positive (presence of any stage of cirrhosis). The exact 95% confidence intervals were computed for AUC, accuracy, sensitivity, and specificity metrics at the patient level (Pearson-Klopper method).

To explore patterns in patient subgroups, subgroup analysis was performed on the final ensemble model with respect to age, BMI, and sex at the patient level.

Model development and hyperparameter tuning were performed using Tensorflow version 2.3.0 for Python version 3.7.8. Data summaries, statistical analysis, visualizations, and model evaluation were performed using R Statistical Software (version 4.0.3); R Foundation for Statistical Computing, Vienna, Austria.

## Results

### Patient demographics

A total of 46 individuals (157 samples) were included in this study (123 samples from 35 patients with decompensated or compensated cirrhosis and 34 control samples from 11 healthy individuals). Among the 46 patients included, median age was 57 (Range: 24–76), 35/46 (76%) had history of liver cirrhosis, 23/46 (50%) were men. A comparison of demographics between healthy and cirrhosis patients is depicted in Table 1. In comparison to healthy controls, cirrhosis patients had an older age at diagnosis (median: 61 vs. 45 years, *P* = 0.001) and were more likely to be obese (51.3% vs. 27.3%).

**TABLE 1:** Comparison of demographics between healthy and cirrhosis patients.

	Median (minimum, maximum) or No. (%) of patients		
	Disease (*N* = 35)	Healthy (*N* = 11)	*P*-value
Sex (Male)			1.00
Female	17 (48.6%)	6 (54.5%)	
Male	18 (51.4%)	5 (45.5%)	
Age (years)	61.0 (33.0, 76.0)	45.0 (24.0, 60.0)	**<0.001**
Age group (years)			**0.002**
(20, 50)	7 (20.0%)	8 (72.7%)	
(50, 80)	28 (80.0%)	3 (27.3%)	
Body mass index (kg/m^2^)	30.2 (20.2, 41.3)	27.6 (21.0, 41.8)	0.42
Body mass index (categorical)			0.20
Healthy weight (18.5–24.9)	5 (14.3%)	2 (18.2%)	
Overweight (25.0–29.9)	10 (28.6%)	6 (54.5%)	
Obesity (>30.0)	20 (57.1%)	3 (27.3%)	

*P*-values result from a Wilcoxon rank sum test (continuous variables) or Fisher’s exact test (categorical variables). Bold values denote statistical significance at the *p* < 0.05 level.

Within the disease cohort, 14 patients (35.9%) had stage I cirrhosis, 15 patients (38.5%) had stage II cirrhosis, and 10 patients (25.6%) had stage III cirrhosis. Two persons with stage III cirrhosis had a history of hepatic encephalopathy that was well controlled and not clinically manifest at time of collection. A comparison of laboratory test results across cirrhosis stages is shown in Table 2. As expected by the cirrhosis classifications, stage III cirrhosis had highest model for end-stage liver disease (MELD), aspartate aminotransferase to platelet ratio index (APRI), and Fibrosis-4 index for liver fibrosis (FIB-4) scores with medians 13, 0.9, 6, respectively. Supplementary Table 1 expands upon Table 2, including additional laboratory test results.

**TABLE 2:** Comparison of characteristics across disease stage for the cirrhosis study population.

	Median (minimum, maximum) or No. (%) of patients			
	Cirrhosis stage I, compensated (*N* = 13)	Cirrhosis stage II, compensated (*N* = 12)	Cirrhosis stage III, decompensated (*N* = 10)	*P*-value
Ascites	0 (0.0%)	0 (0.0%)	10 (100.0%)	**<0.001**
Varices	0 (0.0%)	12 (100.0%)	8 (80.0%)	**<0.001**
Platelets	185.0 (123.0, 272.0)	92.5 (44.0, 279.0)	83.0 (36.0, 238.0)	**0.014**
MELD	8.0 (6.0, 20.0)	10.0 (7.0, 19.0)	13.0 (7.0, 28.0)	**0.041**
APRI	0.4 (0.2, 1.1)	0.8 (0.2, 3.5)	0.9 (0.3, 3.5)	0.16
FIB4	2.4 (0.6, 4.2)	3.7 (1.2, 10.7)	6.0 (1.3, 14.8)	**0.013**
Etiology				0.13
Non-alcoholic steatohepatitis (NASH)	10 (76.9%)	8 (66.7%)	3 (30.0%)	
Alcoholic liver cirrhosis (ALC)	0 (0.0%)	2 (16.7%)	0 (0.0%)	
Hepatitis C Virus (HCV)	1 (7.7%)	1 (8.3%)	1 (10.0%)	
HCV + ALC	0 (0.0%)	0 (0.0%)	2 (20.0%)	
Primary sclerosing cholangitis 2 (PSC 2)	2 (15.4%)	1 (8.3%)	3 (30.0%)	
Hemochromatosis	0 (0.0%)	0 (0.0%)	1 (10.0%)	

*P*-values result from a Kruskal-Wallis rank sum test (continuous variables) or Fisher’s exact test (categorical variables). MELD, model for end-stage liver disease; APRI, aspartate aminotransferase to platelet ratio index; FIB4, Fibrosis-4 index for liver fibrosis. Bold values denote statistical significance at the *p* < 0.05 level.

### Model performance at the sample and patient levels

**FIGURE 4:**
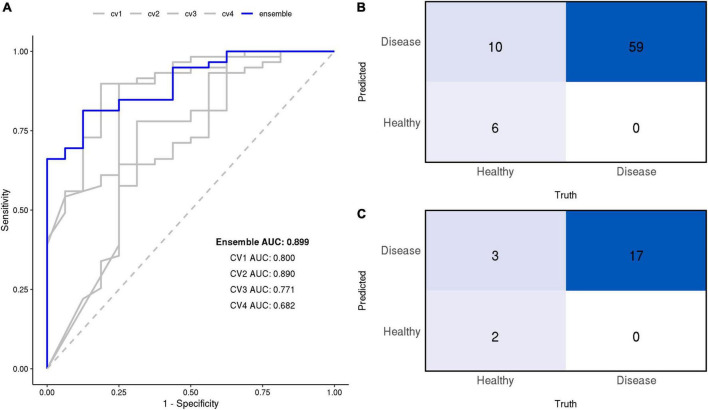
**(A)** Receiver operating characteristic (ROC) curves of the final models at the sample level. Area under the ROC curve (AUC) is annotated for each model. The ensemble’s confusion matrix heatmaps at the sample **(B)** and patient **(C)** levels summarize the frequency of True Positives (TP), False Negatives (FN), True Negatives (TN), False Positives (FP).

The CNN model was successful in differentiating breath samples taken from patients with cirrhosis vs. healthy controls; four models trained on separate CV splits classified the presence of cirrhosis with an average AUC of 0.79 at the sample level (clustering between technical replicates precludes accurate estimation of the exact 95% CI at the sample level; these values are reported for the primary endpoint of patient diagnosis only). When these models were combined into an ensemble by averaging prediction probabilities, the AUC was 0.90 as depicted by Figure 4.

At the patient level, the ensemble model prediction outperformed the four constituent CV models in detecting the presence of cirrhosis in patients. Individual models discriminated between cirrhosis individuals and healthy controls with an average AUC of 0.80 (range: 0.54, 1.00), their ensemble achieved an AUC of 0.90 (95% CI: 0.75, 1.00). At a 50% classification threshold, the ensemble model yielded the following performance metrics: sensitivity of 1.00 (perfect), specificity of 0.40, positive predicted value of 0.85, negative predicted value of 1.00, and F1 score of 91.9.

All diagnostic performance measures for the ensemble and its constituent CV models are reported in Table 3 at both sample and patient levels.

**TABLE 3:** Model performance metrics at sample and patient levels at the 0.5 threshold.

	AUC (95% CI)	Accuracy (95% CI), fraction	Sensitivity (95% CI), fraction	Specificity (95% CI), fraction	PPV (95% CI), fraction	NPV (95% CI), fraction	F1 score
**Sample level**							
Ensemble	0.899	86.7% 65/75	100.0% 59/59	37.5% 6/16	85.5% 59/69	100.0% 6/6	92.2
CV1	0.800	86.7% 65/75	100.0% 59/59	37.5% 6/16	88.7% 55/62	69.2% 9/13	92.2
CV2	0.890	85.3%64/75	93.2% 55/59	56.2% 9/16	88.7% 55/62	69.2% 9/13	90.9
CV3	0.771	85.3% 64/75	98.3% 58/59	37.5% 6/16	85.3% 58/68	85.7% 6/7	91.3
CV4	0.682	81.3%61/75	93.2% 55/59	37.5% 6/16	84.6% 55/65	60.0% 6/10	88.7
**Patient level**							
Ensemble	0.894 (0.751, 1.000)	86.4% (65.1%, 97.1%) 19/22	100.0% (80.5%, 100.0%) 17/17	40.0% (5.3%, 85.3%) 2/5	85.0% (62.1%, 96.8%) 17/20	100.0% (15.8%, 100.0%) 2/2	91.9
CV1	0.824 (0.627, 1.000)	86.4% (65.1%, 97.1%) 19/22	100.0% (80.5%, 100.0%) 17/17	40.0% (5.3%, 85.3%) 2/5	85.0% (62.1%, 96.8%) 17/20	100.0% (15.8%, 100.0%) 2/2	91.9
CV2	0.882 (0.691, 1.000)	81.8% (59.7%, 94.8%) 18/22	88.2% (63.6%, 98.5%) 15/17	60.0% (14.7%, 94.7%) 3/5	88.2% (63.6%, 98.5%) 15/17	60.0% (14.7%, 94.7%) 3/5	88.2
CV3	0.800 (0.486, 1.000)	86.4% (65.1%, 97.1%) 19/22	100.0% (80.5%, 100.0%) 17/17	40.0% (5.3%, 85.3%) 2/5	85.0% (62.1%, 96.8%) 17/20	100.0% (15.8%, 100.0%) 2/2	91.9
CV4	0.682 (0.371, 0.994)	81.8% (59.7%, 94.8%) 18/22	94.1% (71.3%, 99.9%) 16/17	40.0% (5.3%, 85.3%) 2/5	84.2% (60.4%, 96.6%) 16/19	66.7% (9.4%, 99.2%) 2/3	88.9

95% Confidence Intervals are reported at the patient level only, clustering of technical replicates precluded calculation of the exact confidence interval at the sample level. PPV, positive predictive value; NPV, negative predictive value.

The subgroup analysis did not reveal any significant differences in model performance between subgroups (age, BMI, or sex) indicating that age is not a confounding factor in classification of breath samples.

### Performance based on the cirrhosis stage

**FIGURE 5:**
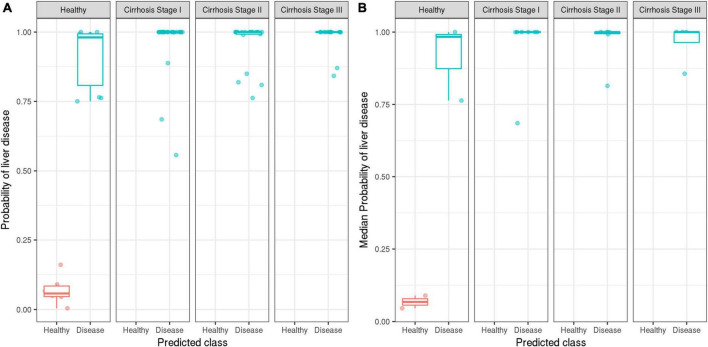
Distribution of the ensemble model’s predicted probabilities for healthy vs. disease classifications stratified by the true stage of cirrhosis. Ground truth labels of healthy (red) and disease (blue) are displayed. On the *y*-axis, probability values of model output are displayed. Model performance is reported at the sample level **(A)**, as well as patient level **(B)** by aggregating based on median probabilities.

At the 50% threshold, the model correctly classified 100% of patients with stage I, stage II, or stage III cirrhosis (17/17 patients; 59/59 samples), i.e., the model achieved perfect sensitivity. The model correctly identified 2/5 healthy individuals (6/16 healthy samples) but incorrectly classified 3/6 healthy individuals (10/16 healthy samples) as having cirrhosis (Figure 4). Evaluation of the ensemble model at classifying the presence or absence of cirrhosis at several stages of cirrhosis is shown (Figure 5). The model displayed higher confidence when the patient had stage II or II cirrhosis (median probabilities > 0.99) than when they had stage I disease (median probability > 0.76).

### Identification of volatile-organic compound features

**FIGURE 6:**
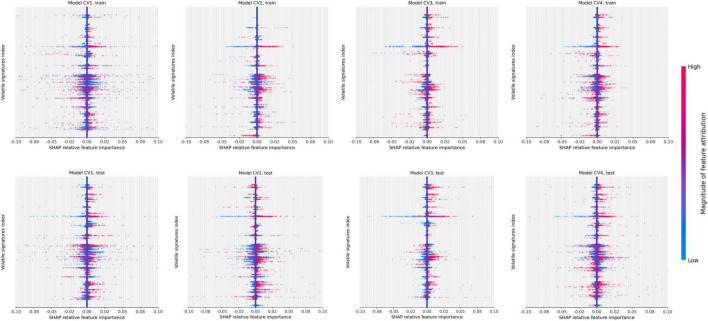
Beeswarm summary plots on train and test data. This plot combines feature importance and feature effects. Every feature (VOC) is represented as a row on the *y*-axis (3,400 total) and SHAP values are on the *x*-axis (multiple VOC may overlap at a single index). Each dot represents a Shapley value for a given sample prediction. The color intensity shows the magnitude of importance of each feature.

**FIGURE 7:**
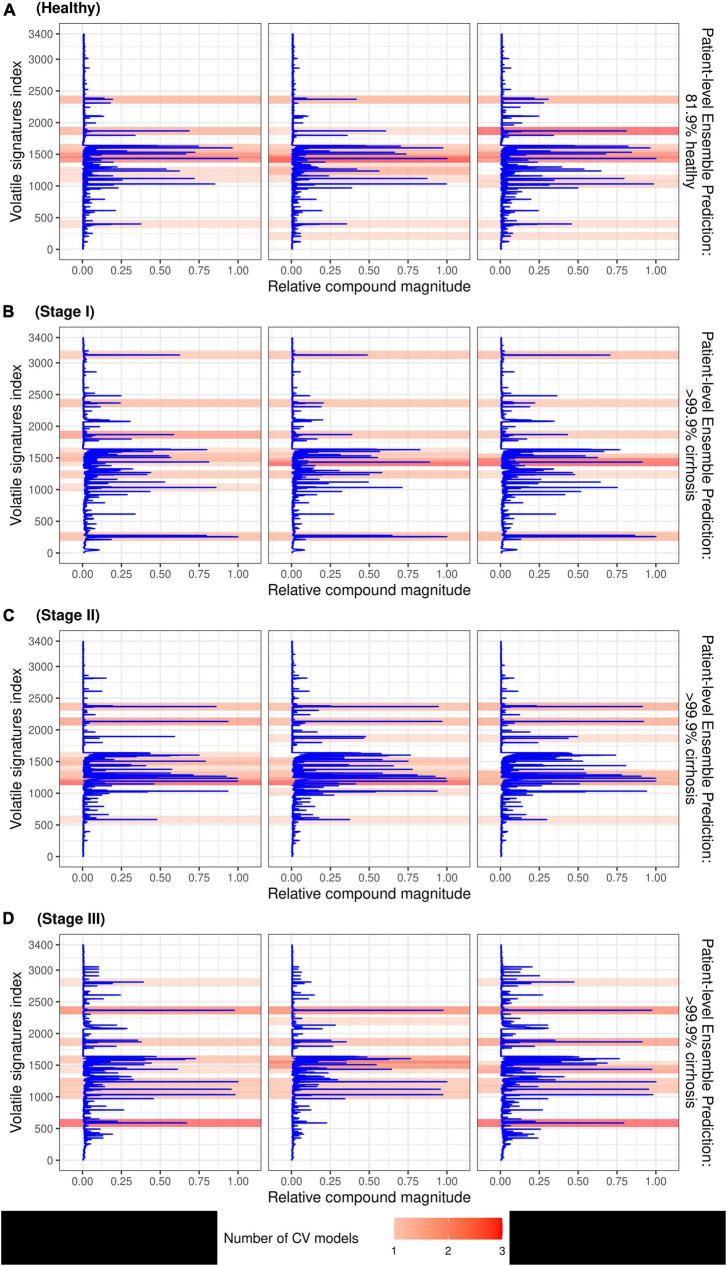
Patient breath samples with overlayed heatmaps which identify the 5 most important peaks from each CV model (up to 20 peaks total) in the classification of liver cirrhosis for a healthy control **(A)**, and 3 individuals with stage I **(B)**, stage II **(C)**, and stage III **(D)** cirrhosis, respectively. Compounds are represented by indices on the *y*-axis and VOC signal value is on the *x*-axis; darker shading indicates the feature was selected by multiple CV models.

The SHAP values which identify peaks in the signal that contributed most to the prediction are depicted by the beeswarm summary plots in Figure 6. For each CV model, we identified the top 10 peaks which selected a total of 22 unique compounds in the TD-GC-FAIMS signal; 14 compounds (64%) were identified by at least two independently trained CV models, two compounds were identified by three CV models, and one compound was identified by all four CV models. Figure 7 displays an example of four patients (one from each stage of cirrhosis and one healthy control) whose VOC profiles’ signal is visualized with overlaying heatmaps, which depict the five most important compounds in the classification of liver cirrhosis identified by each model.

## Discussion

This work presents a deep-learning based approach for detecting liver cirrhosis based on non-invasive breath samples analyzed with TD-GC-FAIMS. To our knowledge, this is the first application of deep-neural networks for the prediction of liver cirrhosis from volatolomic profiles from patient breath samples (25–28). We observed that CNNs were an effective technique for analyzing the volatolomic profiles obtained using TD-GC-FAIMS, and our optimal model displayed an AUC of 0.90 and an accuracy of 86% at the patient level. This supports the application of volatolomic analyses using TD-GC-FAIMS for non-invasive diagnosis of cirrhosis from breath samples.

### Deep learning approach

Several deep-learning approaches were attempted including transfer-learning of a pretrained ResNet, and a fully connected deep neural network. Previous experiments done by this group have demonstrated machine-learning based approaches for the detection of cirrhosis (19). We observed optimal performance with a CNN model. This is consistent with extensive literature that indicates CNNs are an efficient and accurate method of analyzing sparse signals data; in the medical field, CNNs are popular model for both image analysis and signals processing (22, 29–32).

Optimal performance was observed with an ensemble of four CNNs combined by taking the mean prediction probability; the ensemble performance was slightly better than the best performing constituent models, and substantially better than the average of its four constituents. Combining several models into an ensemble is an effective technique for generating consistent predictions and reducing the impact of overfitting.

### Model performance in stage I and stage II cirrhosis

Our model was effective in predicting the presence of cirrhosis with an accuracy of 86% at the patient level. The model displayed a tendency to overdiagnose the presence of cirrhosis; the ensemble model had a sensitivity of 100% but a specificity of 40% at the patient level.

At the sample level, all mistakes came from differentiating healthy controls from patients with stage I cirrhosis (e.g., the lowest stage of disease, when individuals are often asymptomatic). This suggests that the model is correctly identifying hallmarks of advanced cirrhosis with a very high level of accuracy.

Imbalance in the training dataset likely played a role in model specificity (only 11/46 individuals included in this study were healthy controls). Specificity may be modified by adjusting the prediction cutoff from 0.5 to a higher value, with the understanding that this may increase the rate of false negatives. A diagnostic tool with high sensitivity could be appropriate as an inexpensive, non-invasive screening tool for cirrhosis detection in an at-risk population, with the understanding that additional diagnostic tests such as imaging exams would be required to rule out false positive results in an initial screen.

### Explainable artificial intelligence

The use of SHAP for explaining the predictions of the CNN model identified several discrete peaks which were consistently associated with either a positive or negative prediction. We observed that 14/22 (63%) of the top peaks detected by the ensemble model were identified by multiple independent CV models, which indicates that these features are reproducible between independently trained models. This supports the reliability of the CNN approach.

Several specific VOCs are known to be overexpressed or underexpressed in cirrhotic patients, including limonene, methanol, and 2-pentanone (8). Data-driven approaches such as deep neural networks rely on the entire volatolomic profile measurements, not only a few discrete peaks, and therefore may be incorporating VOCs which have not yet been identified, or VOC constituents which are partially metabolized from known compounds. Future work has the potential to characterize previously unknown VOCs which the model indicates are implicated in cirrhosis.

### Limitations

We acknowledge several important limitations to this study. Although this is a preliminary study in a relatively modest dataset of 46 patients (157 samples) with unbalanced groups, several observations strongly support the conclusion that the model is capturing a true volatolomic signature which can diagnose disease. Firstly, all four crossvalidated models demonstrated strong predictive performance on an independent test dataset of 22 patients that were not seen at any point in the model training and validation process, and therefore is likely not the result of overfitting (AUC 0.682–0.882). Secondly, model confidence correlated to cirrhosis stage (median probabilities > 0.99 for Stage II, Stage III cirrhosis, median probability > 0.76 for Stage I, healthy) which is consistent with the clinical observation that it is more difficult to detect lower grade cirrhosis; furthermore, subgroup analysis did not indicate any confounding with age or sex. Thirdly, SHAP analysis identified 64% of features were identified by at least two independently trained CV models; the model is consistently identifying several discrete features in multiple patient samples. Further experimental work is needed to identify which specific compounds are identified by these peaks.

Ongoing subject recruitment focuses on the collection of additional samples, but reporting of findings on the initial dataset is required to demonstrate proof-of-concept, and to support the expensive and labor-intensive collection of additional samples, as well as to justify the recruitment of additional patient participants.

## Conclusion

A deep learning model is capable of detecting the presence of cirrhosis in volatolomic profiles obtained from analyses of exhaled breath samples from patients using TD-GC-FAIMS. Model performance had an AUC of 0.90 and a sensitivity in detecting disease of 100% at the patient level. Use of SHAP as a technique for explainable AI detected a set of unique peaks associated with both positive and negative prediction; 64% of the top 10 peaks were reproducible across multiple independently trained models. This technique demonstrates feasibility of a non-invasive clinical screening exam for diagnosing and monitoring liver cirrhosis from non-invasive breath samples without the need for detection and characterization of individual metabolites.

## Data availability statement

The raw data supporting the conclusions of this article will be made available by the authors, without undue reservation.

## Ethics statement

The studies involving human participants were reviewed and approved by the Mayo Clinic Institutional Review Board. Written informed consent for participation was not required for this study in accordance with the national legislation and the institutional requirements.

## Author contributions

ML and JT performed the experimental work and reviewed the manuscript. MW and AW performed the data analysis and modeling and drafted the manuscript. RC and TP supervised the work and reviewed the manuscript. All authors contributed to the article and approved the submitted version.
