# Supplementary figures and images for: A deep learning approach for detecting liver cirrhosis from volatolomic analysis of exhaled breath
Source: Front Med (Lausanne). 2022 Sep 29;9:992703. doi: 10.3389/fmed.2022.992703 (PMC9556819; doi:10.3389/fmed.2022.992703)

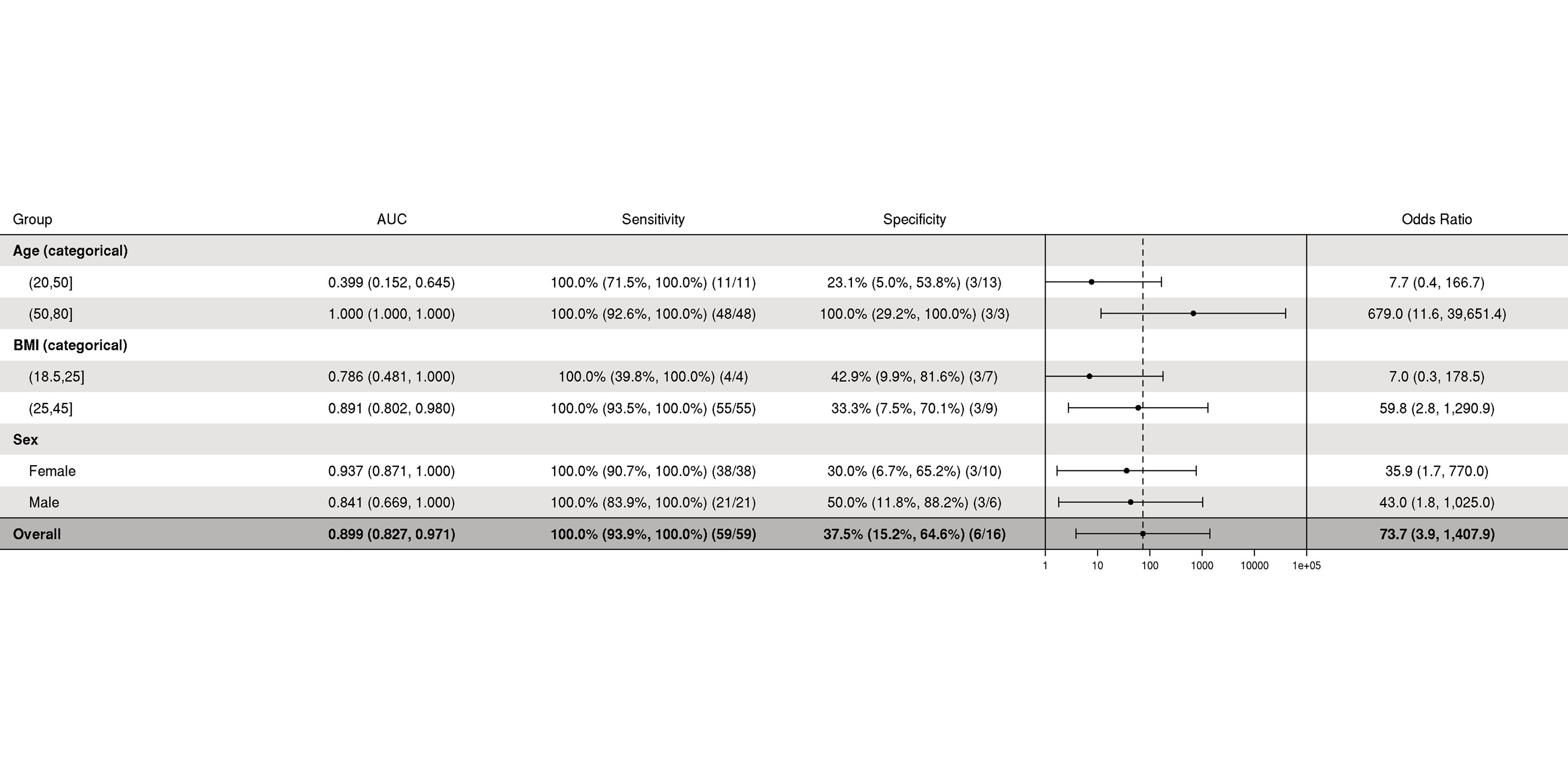

Supplement: Supplementary file 1 [file Image_1.jpg]
