# Supplementary material for: A deep learning approach for detecting liver cirrhosis from volatolomic analysis of exhaled breath
Source: Front Med (Lausanne). 2022 Sep 29;9:992703. doi: 10.3389/fmed.2022.992703 (PMC9556819; doi:10.3389/fmed.2022.992703)
Supplement: Supplementary file 2 [file Table_1.docx]

**Supplemental Table 1. Comparison of characteristics across disease stage for the cirrhosis study population.**

|  | | Median (minimum, maximum) or No. (%) of patients | | |
| --- | --- | --- | --- | --- |
|  | N | Cirrhosis Stage I, Compensated (N=13) | Cirrhosis Stage II, Compensated (N=12) | Cirrhosis Stage III, Decompensated (N=10) |
| Ascites | 35 | 0 (0.0%) | 0 (0.0%) | 10 (100.0%) |
| Varices | 35 | 0 (0.0%) | 12 (100.0%) | 8 (80.0%) |
| Platelets | 35 | 185.0 (123.0, 272.0) | 92.5 (44.0, 279.0) | 83.0 (36.0, 238.0) |
| MELD | 35 | 8.0 (6.0, 20.0) | 10.0 (7.0, 19.0) | 13.0 (7.0, 28.0) |
| APRI | 35 | 0.4 (0.2, 1.1) | 0.8 (0.2, 3.5) | 0.9 (0.3, 3.5) |
| FIB4 | 35 | 2.4 (0.6, 4.2) | 3.7 (1.2, 10.7) | 6.0 (1.3, 14.8) |
| Etiology | 35 |  |  |  |
| Nonalcoholic Steatohepatitis (NASH) |  | 10 (76.9%) | 8 (66.7%) | 3 (30.0%) |
| Alcoholic liver cirrhosis (ALC) |  | 0 (0.0%) | 2 (16.7%) | 0 (0.0%) |
| Hepatitis C Virus (HCV) |  | 1 (7.7%) | 1 (8.3%) | 1 (10.0%) |
| HCV+ALC |  | 0 (0.0%) | 0 (0.0%) | 2 (20.0%) |
| Primary sclerosing cholangitis 2 (PSC 2) |  | 2 (15.4%) | 1 (8.3%) | 3 (30.0%) |
| Hemochromatosis |  | 0 (0.0%) | 0 (0.0%) | 1 (10.0%) |
| Total Bilirubin | 35 | 0.8 (0.2, 4.3) | 1.0 (0.5, 9.2) | 1.0 (0.4, 2.4) |
| AST | 35 | 39.0 (24.0, 66.0) | 33.5 (25.0, 188.0) | 36.5 (20.0, 116.0) |
| ALT | 35 | 41.0 (20.0, 81.0) | 34.0 (21.0, 357.0) | 28.0 (20.0, 78.0) |
| Alkaline Phosphatase | 35 | 110.0 (45.0, 158.0) | 93.0 (71.0, 1612.0) | 113.5 (39.0, 247.0) |
| Creatine | 35 | 0.8 (0.5, 1.9) | 0.8 (0.4, 1.0) | 0.9 (0.6, 4.1) |
| Serum Sodium | 35 | 140.0 (135.0, 144.0) | 139.5 (136.0, 142.0) | 138.0 (127.0, 143.0) |
| Albumin | 35 | 4.2 (2.7, 4.7) | 4.0 (3.5, 4.4) | 3.8 (3.1, 4.8) |
| AFP | 21 | 3.1 (1.3, 6.7) | 3.0 (1.8, 7.7) | 2.5 (1.2, 5.3) |
| Leukocytes | 35 | 5.3 (3.4, 10.5) | 4.9 (1.8, 9.9) | 3.8 (1.7, 7.0) |
| Hemoglobin | 35 | 12.9 (9.8, 14.9) | 12.9 (11.0, 16.1) | 11.6 (7.4, 16.1) |
| INR | 35 | 1.1 (0.8, 1.8) | 1.2 (1.1, 1.5) | 1.3 (0.9, 2.7) |
| Abbreviations: MELD = model for end-stage liver disease, APRI = aspartate aminotransferase to platelet ratio index, FIB4 = Fibrosis-4 index for liver fibrosis, AST = aspartate aminotransferase, ALT = alanine transaminase, AFP = alpha-fetoprotein, INR = international normalized ratio. | | | | |
