# Supplementary material for: A deep learning approach for detecting liver cirrhosis from volatolomic analysis of exhaled breath
Source: Front Med (Lausanne). 2022 Sep 29;9:992703. doi: 10.3389/fmed.2022.992703 (PMC9556819; doi:10.3389/fmed.2022.992703)
Supplement: Supplementary file 3 [file Table_2.docx]

**Supplemental Table 2. Number (percentage) of samples and patients in each Cross-Validation (CV) split.**

|  | **Disease** | **Healthy** | **Total** |
| --- | --- | --- | --- |
| **Sample level** | N=123 | N=34 | N=157 |
| Test | 59 (48.0%) | 16 (47.1%) | 75 (47.8%) |
| Train | 64 (52.0%) | 18 (52.9%) | 82 (52.2%) |
| CV Split 1 |  |  |  |
| - Analysis | 50 (78.1%) | 12 (66.7%) | 62 (75.6%) |
| - Assessment | 14 (21.9%) | 6 (33.3%) | 20 (24.4%) |
| CV Split 2 |  |  |  |
| - Analysis | 42 (65.6%) | 13 (72.2%) | 55 (67.1%) |
| - Assessment | 22 (34.4%) | 5 (27.8%) | 27 (32.9%) |
| CV Split 3 |  |  |  |
| - Analysis | 50 (78.1%) | 14 (77.8%) | 64 (78.0%) |
| - Assessment | 14 (21.9%) | 4 (22.2%) | 18 (22.0%) |
| CV Split 4 |  |  |  |
| - Analysis | 50 (78.1%) | 15 (83.3%) | 65 (79.3%) |
| - Assessment | 14 (21.9%) | 3 (16.7%) | 17 (20.7%) |
| **Patient level** | N=35 | N=11 | N=46 |
| Test | 17 (48.6%) | 5 (45.5%) | 22 (47.8%) |
| Train | 18 (51.4%) | 6 (54.5%) | 24 (52.2%) |
| CV Split 1 |  |  |  |
| - Analysis | 14 (77.8%) | 4 (66.7%) | 18 (75.0%) |
| - Assessment | 4 (22.2%) | 2 (33.3%) | 6 (25.0%) |
| CV Split 2 |  |  |  |
| - Analysis | 12 (66.7%) | 4 (66.7%) | 16 (66.7%) |
| - Assessment | 6 (33.3%) | 2 (33.3%) | 8 (33.3%) |
| CV Split 3 |  |  |  |
| - Analysis | 14 (77.8%) | 5 (83.3%) | 19 (79.2%) |
| - Assessment | 4 (22.2%) | 1 (16.7%) | 5 (20.8%) |
| CV Split 4 |  |  |  |
| - Analysis | 14 (77.8%) | 5 (83.3%) | 19 (79.2%) |
| - Assessment | 4 (22.2%) | 1 (16.7%) | 5 (20.8%) |
