# Supplementary material for: A deep learning approach for detecting liver cirrhosis from volatolomic analysis of exhaled breath
Source: Front Med (Lausanne). 2022 Sep 29;9:992703. doi: 10.3389/fmed.2022.992703 (PMC9556819; doi:10.3389/fmed.2022.992703)
Supplement: Supplementary file 4 [file Table_3.docx]

**Supplemental Table 3. Hyperparameter configuration for optimal model**

| Hyperparameter | Value |
| --- | --- |
| Learning Rate | 0.005 |
| Learning Rate Optimizers | Adam [32],  Reduce LR by 0.5 after 10 epoch plateau |
| Batch Size | 5 |
| Loss | Categorical cross-entropy |
| Regularization | L2 (alpha = 0.01)  Batch normalization  (momentum = 0.99, epsilon=0.001) |
| Training epochs | 200 |
| Validation | 4-fold cross validation |
|  |  |
